# The effect of spike mutations on SARS-CoV-2 neutralization

**DOI:** 10.1016/j.celrep.2021.108890

**Published:** 2021-03-06

**Authors:** Chloe Rees-Spear, Luke Muir, Sarah A. Griffith, Judith Heaney, Yoann Aldon, Jonne L. Snitselaar, Peter Thomas, Carl Graham, Jeffrey Seow, Nayung Lee, Annachiara Rosa, Chloe Roustan, Catherine F. Houlihan, Rogier W. Sanders, Ravindra K. Gupta, Peter Cherepanov, Hans J. Stauss, Eleni Nastouli, on behalf of the SAFER Investigators, Katie J. Doores, Marit J. van Gils, Laura E. McCoy

**Affiliations:** 1 Institute of Immunity and Transplantation, Division of Infection and Immunity, University College London, London NW3 2PF, UK; 2 Advanced Pathogens Diagnostic Unit, Department of Clinical Virology, University College London Hospitals NHS Foundation Trust, London W1T 4EU, UK; 3 Amsterdam University Medical Centers, Amsterdam Institute for Infection and Immunity, University of Amsterdam, 1105 AZ Amsterdam, the Netherlands; 4 School of Immunology and Microbial Sciences, King’s College London, London SE1 9RT, UK; 5 The Francis Crick Institute, London NW1 1AT, UK; 6 Research Department of Infection, Division of Infection and Immunity, University College London, London WC1 6BT, UK; 7 Department of Medicine, University of Cambridge, Cambridge CB2 0AW, UK; 8 Great Ormond Street Institute for Child Health, Infection, Immunity and Inflammation, University College London, London WC1N 1EH, UK

**Keywords:** SARS-CoV-2, neutralization, antibodies, serology, immune escape, variant, B.1.1.7

## Abstract

Multiple severe acute respiratory syndrome coronavirus 2 (SARS-CoV-2) vaccines show protective efficacy, which is most likely mediated by neutralizing antibodies recognizing the viral entry protein, spike. Because new SARS-CoV-2 variants are emerging rapidly, as exemplified by the B.1.1.7, B.1.351, and P.1 lineages, it is critical to understand whether antibody responses induced by infection with the original SARS-CoV-2 virus or current vaccines remain effective. In this study, we evaluate neutralization of a series of mutated spike pseudotypes based on divergence from SARS-CoV and then compare neutralization of the B.1.1.7 spike pseudotype and individual mutations. Spike-specific monoclonal antibody neutralization is reduced dramatically; in contrast, polyclonal antibodies from individuals infected in early 2020 remain active against most mutated spike pseudotypes, but potency is reduced in a minority of samples. This work highlights that changes in SARS-CoV-2 spike can alter neutralization sensitivity and underlines the need for effective real-time monitoring of emerging mutations and their effect on vaccine efficacy.

## Highlights

• SARS-CoV-2 pseudotypes produced by amino acids substitutions in SARS-CoV
• SARS-CoV-2 pseudotyped virus that encodes the B.1.1.7 variant spike
• Amino acid changes and B.1.1.7 can decrease monoclonal antibody neutralization
• Minimal effect on sera with only 10% losing potency against the B.1.1.7 pseudotype

## Graphical abstract

**Figure:**
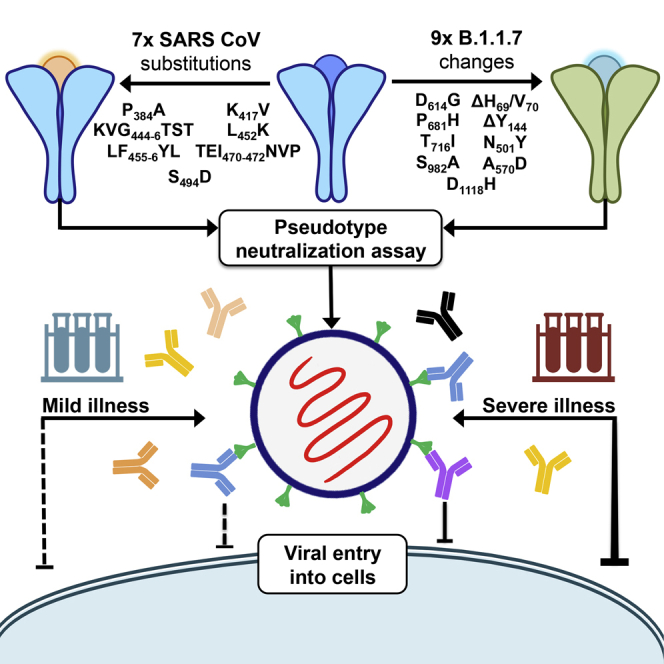

## Teaser

This study describes neutralization by antibodies and convalescent sera of SARS-CoV-2 spike mutants. Rees-Spear et al. show that SARS-CoV amino acid substitutions and the B.1.1.7 variant can block monoclonal antibody neutralization and that serum samples collected following mild illness are less resilient to spike variation than those following severe illness.

## Introduction

Serum neutralization activity is a common correlate of protection against viral infection following vaccination or natural infection (Plotkin, 2008). However, effective protection from viral infection can also require a sufficient breadth of serum neutralization rather than potency alone. This is because of the high levels of variation observed in major antigens across some viral populations (Burton et al., 2012). For example, in the response against influenza, the majority of neutralizing serum antibodies target only a particular set of influenza strains as a result of antigenic drift of the immunodominant hemagglutinin head (Zost et al., 2019). Because of this, an annual vaccine is required and must be matched to the most probable circulating strain in any given year to ensure protection from infection. Data emerging from human vaccine trials and challenge studies in animal models suggest that neutralizing antibodies can prevent disease caused by infection with severe acute respiratory syndrome coronavirus 2 (SARS-CoV-2), the virus that causes coronavirus disease 2019 (COVID-19) (McMahan et al., 2021; Polack et al., 2020). However, new variants of SARS-CoV-2 have begun to emerge (Kemp et al., 2020; Oude Munnink et al., 2021; Tegally et al., 2020; Welkers et al., 2021). These variants include mutations in the major neutralizing antigen, the spike glycoprotein, and raises the question of whether neutralizing serum responses induced by early circulating strains or by vaccines based on the spike sequence of these early strains can neutralize the recently emerged virus variants.

Prior to emergence of multiple mutations in spike in the human population, we reasoned that a logical way to identify potential escape mutations was to look at sites of amino acid variation relative to the most closely related human betacoronavirus, SARS-CoV, which caused the original SARS outbreak (CDC, 2003). These two closely related viruses are characterized by notable differences in transmission dynamics and disease outcomes (Cevik et al., 2020; Lipsitch et al., 2003; Petersen et al., 2020), but use the human ACE2 protein as a viral entry receptor (Li et al., 2003) and share approximately 75% similarity overall in spike at the amino acid level (Gralinski and Menachery, 2020). Both viruses use the same region of their respective spikes to bind ACE2, the receptor binding domain (RBD; found in the S1 subunit of spike). There is considerable amino acid variation between the two RBDs despite their conserved binding to ACE2, which explains why the majority of COVID-19 sera have weaker or no neutralizing activity against SARS-CoV, but cross-neutralizing monoclonal antibodies (mAbs) have been isolated (Brouwer et al., 2020).

Since the start of the pandemic, sequencing of virus populations has been deployed to enable detection of individual mutations in SARS-CoV-2. Recently, a new variant, B.1.1.7, has emerged in the United Kingdom (Kemp et al., 2020; Rambaut et al., 2020) that includes multiple mutations in the RBD and the N-terminal domain (NTD) of spike, targets for neutralizing antibodies. Similarly, additional variants have been identified in South Africa (B.1.351) and Brazil (P.1) (Faria et al., 2021; Tegally et al., 2020). The B.1.351 and P.1 variants share a deletion of three amino acids in Orf1ab and key mutations in the RBD (E484K and N501Y); data so far consistent with convergent evolution and recombination (Varabyou et al., 2020). Early reports indicated that, although the RBD mutation N501Y in the B.1.1.7 strain does not compromise post-vaccine serum neutralization (Xie et al., 2021), the additional changes in the B.1.351 strain do impair neutralization (Greaney et al., 2021; Wibmer et al., 2021).

In this study, we evaluated the potential role of individual amino acids in facilitating escape from neutralizing antibodies. First, we made a series of point mutations to change the amino acids in SARS-CoV-2 to those found at the analogous position in SARS-CoV. Second, we made individual point mutations emerging in real-world populations and generated a pseudotype virus using the B.1.1.7 variant spike sequence. We identify multiple mutations that can abrogate neutralization by some mAbs targeting the RBD of spike. However, in contrast, we show that serum responses are more resilient to these mutations, especially following severe illness, where the antibody response is characterized by increased breadth.

## Results

### Generation of potential escape mutants by SARS-CoV amino acid substitution

There are 56 individual amino acid changes between the RBD of SARS-CoV-2 and SARS-CoV (Ortega et al., 2020). We prioritized 15 of the 56 changes by considering which mutations resulted in amino acids of substantially different biochemical character and which changes occurred in sequential positions. These sites were mutated in the SARS-CoV-2 spike to match SARS-CoV (Figure S1) and used to produce pseudotyped viruses (Seow et al., 2020). Twelve of the 15 mutated pseudotypes gave virus titers and were then screened for any alteration in neutralization against a panel of human mAbs (Brouwer et al., 2020) isolated after SARS-CoV-2 infection. These mAbs have been mapped previously into 11 binding clusters, where mAbs within a cluster compete reciprocally for binding to spike. Representatives of each neutralizing cluster were selected for evaluation against the spike mutant pseudotypes.

### Effect of SARS-CoV spike substitutions on SARS-CoV-2 mAb neutralization

**Figure 1:**
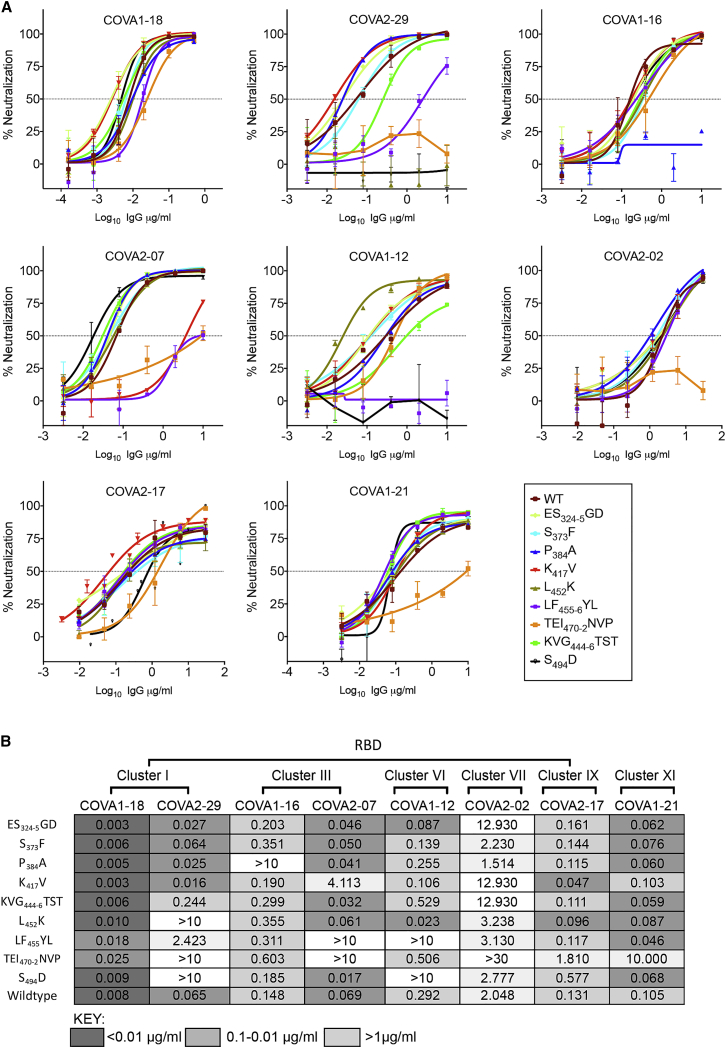
Mutating amino acids in SARS-CoV-2 spike to match SARS-CoV decreases mAb neutralization (A) The indicated mAbs were assessed by pseudotyped neutralization assay. Data are representative of three independent repeats. The horizontal dotted line in each graph indicates 50% neutralization. (B) IC_50_ values for each mAb against the mutant SARS-CoV-2 pseudotyped viruses indicated in the left column. The previously established binding cluster and binding to RBD for each mAb are indicated above each column. See also Figure S1.

Initial screening assays showed no major effect on neutralization by any of the mAbs against pseudotypes encoding RFA_346-8_KFP, S_459_G, and ST_477-8_GK. In contrast, the remaining nine viral pseudotype mutants diminished neutralization for at least one mAb, as described below (Figures 1A and 1B).

#### P_384_A

The P_384_A substitution resulted in complete loss of neutralization by COVA1-16, a cluster III RBD-specific mAb that allosterically competes with ACE2 rather than directly blocking the binding site (Liu et al., 2020). This mutation has been described and characterized structurally elsewhere (Wu et al., 2020), revealing that this proline-to-alanine change results in a relatively small alteration in protein structure that can enable SARS-CoV mAbs to neutralize SARS-CoV-2 P_384_A. However, P_384_A does not weaken neutralization by any other mAbs, including another mAb in the cluster III competition group.

#### K_417_V

The K_417_V mutation results in a pseudotyped virus that is less susceptible to COVA2-07-mediated RBD-specific neutralization. That this mutation should affect this mAb, which competes directly with ACE2 for binding, is not unexpected because the lysine at position 417 forms a hydrogen bond with ACE2 (Lan et al., 2020) that is likely disrupted by this substitution. We then evaluated an additional mAb, COVA2-04, from the same competitive binding cluster as COVA2-07. This is because COVA2-04 is representative of a class of SARS-CoV-2-neutralizing antibodies that use the VH3-53 gene (Cao et al., 2020; Mor et al., 2020; Robbiani et al., 2020). COVA2-04 was not able to neutralize the K_417_V pseudotype (data not shown).

#### KVG_444-6_TST

This multiple substitution, which is a substantial change between SARS-CoV-2 and SARS-CoV, results in a 3.7-fold drop in neutralization potency for COVA2-29, which is a cluster I RBD-specific antibody. This is the largest effect of this mutation; the neutralization activity of the other mAbs is largely unaffected despite alteration of three sequential amino acids. This may be explained by the relatively minor differences in the amino acid side chains at the mutated residues.

#### L_452_K

This mutation is situated directly in the receptor binding motif (RBM) of the RBD. It renders pseudotyped virus resistant to neutralization by COVA2-29 but does not affect the other cluster I mAb COVA1-18 or any other mAb tested.

#### LF_455-6_YL

This double substitution reduces neutralization by RBD-specific mAbs from different clusters; specifically, the cluster I mAb COVA2-29, cluster III mAb COVA2-07, and cluster VI mAb COVA1-12. For COVA1-12, all neutralization activity is abolished, whereas COVA2-07 activity is just below the level required to calculate a 50% inhibitory concentration (IC_50_).

#### TEI_470-2_NVP

This triple mutation is located in a loop within the RBM where other substitutions have been reported to abolish ACE2 binding (Xu et al., 2021; Yi et al., 2020). This mutation prevents neutralization by COVA2-29 (cluster I), COVA2-07 (cluster III), and COVA2-02 (cluster VII). It also reduces the activity of the most potent mAb, COVA1-18 (cluster I), whereas this mAb is only minimally affected by other mutations. Moreover, TEI_470-2_NVP lowers the potency of the structurally unmapped non-RBD cluster XI mAb, COVA1-21, to the limit of detection.

#### S_494_D

This single substitution toward the end of the RBM destroys neutralization activity by COVA2-29 (cluster I) and COVA1-12 (cluster VI) but does not have a major effect on the other cluster I mAbs tested or those from other epitope clusters.

In summary, different mAbs can lose their neutralization activity when confronted with different spike mutations, and the effects are not delineated strictly by binding clusters, so mAbs in the same competition cluster are frequently affected differentially. The triple substitution TEI_470-2_NVP has the most detrimental effect on different antibodies and affects mAbs from nearly all binding clusters, and S_494_D also affects many different clusters.

### Effect of spike mutations on serum neutralization

**Figure 2:**
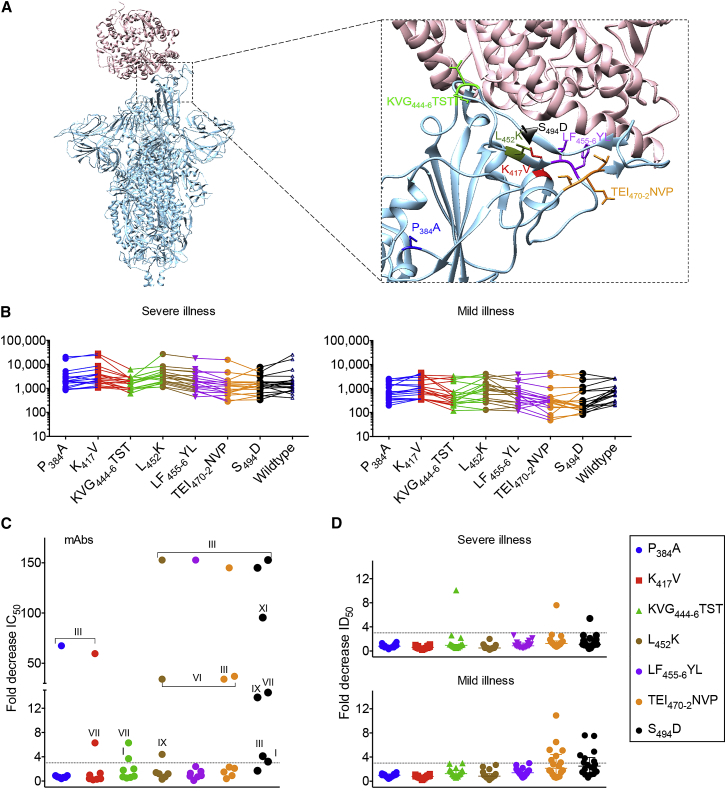
Neutralization by serum is affected less adversely by SARS-CoV amino acid substitutions in SARS-CoV-2 spike (A) Representation of the SARS CoV-2 spike trimer (blue) in complex with ACE-2 (pink) (PDB: 7DF4). The magnified image shows mutated amino acid side chains at residues of interest. (B) Thirty-six serum samples were assessed by pseudotyped neutralization assay. Average ID_50_ values for 3 independent repeats are linked by horizontal bars for each individual sample. (C) Fold decrease in IC_50_ values for each mAb against each mutant pseudotype relative to the SARS-CoV-2 wild-type pseudotype. Competitive binding clusters of each mAb that loses more than 3-fold neutralization activity are labeled. (D) The y axis shows the fold decrease in ID_50_ values for each serum sample against each mutant pseudotype relative to the SARS-CoV-2 wild-type pseudotype; the group of affected individuals is indicated above each graph. (C and D) The dotted horizontal lines indicate a 3-fold drop in neutralization potency.

Following identification of seven spike mutations that can limit or abrogate neutralizing activity of mAbs (Figure 2A), the next step was to assess the effect of these mutations on serum neutralization. Samples were tested following two different scenarios: from a previously characterized cohort of healthcare workers who experienced mild COVID-19 (Houlihan et al., 2020) and sera from a cohort of hospitalized individuals who experienced severe COVID-19. Samples from both cohorts were collected between March and July 2020. Eighteen samples were chosen from both cohorts to obtain representatives with intermediate (1:100–1,000) and potent (>1:1,000) neutralizing 50% inhibitory dilution (ID_50_) values (Figure 2B). Strikingly, serum samples from both cohorts are less affected by spike mutations than individual mAbs in terms of fold decrease in neutralization potency (Figures 2C and 2D). Only one of 36 serum samples lost all neutralizing activity, in contrast to the five mAbs from five different epitope clusters where neutralization was abrogated completely by a single spike mutation (Figure 1C). Moreover, the fold decrease in neutralization potency was more modest for sera than mAbs, with an average 3-fold decrease across all sera for the most disadvantageous mutation, TEI_470-2_NVP, compared with a more than 100-fold decrease observed for several of the mAbs (Figures 2C and 2D). Interestingly, only one of the 36 serum samples lost more than 3-fold potency against the other triple substitution, KVG_444-6_TST, which is consistent with recent data showing that a single mutation at G_446_ caused a major loss of neutralization in one sample (Greaney et al., 2021). Importantly, there was a notable difference between the resilience of serum samples from severely ill, hospitalized individuals and those who experienced mild illness. Only three serum samples from hospitalized individuals lost more than 3-fold potency against any individual mutant (Figure 2D), whereas half of the mild illness serum samples showed a 3-fold drop in potency against at least one spike mutant (Figure 2D).

### Greater levels of spike-reactive antibodies in sera after severe illness

**Figure 3:**
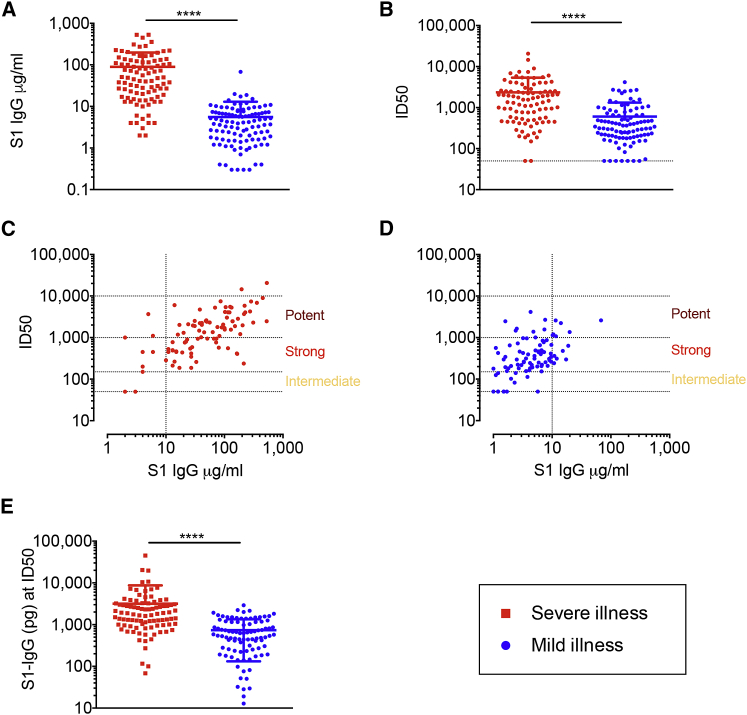
Serum responses following severe COVID-19 have greater polyclonality but less efficient neutralization (A) The y axis shows spike S1 subunit semiquantitative titers measured by ELISA (STAR Methods). (B) The y axis shows ID_50_ values measured by pseudotyped neutralization assay. (C and D) ID_50_ values for serum samples versus the corresponding S1 IgG binding titer. The relative ranking of neutralization titers is indicated in the graph. (E) Concentrations of S1-specific serum IgG (picograms) at ID_50_ dilutions were calculated using the IgG titers quantified via semiquantitative ELISA. Data for (A), (B), and (E) were analyzed by a non-parametric Mann-Whitney *U* test; ∗∗∗∗p < 0.05. Data were measured in duplicate. Mild and severe illness groups are defined in STAR Methods. See also Figure S2.

The differences in resilience to spike mutations seen in the neutralizing sera from these two infection scenarios is plausibly due to greater polyclonality arising from greater antigenic stimulation during severe illness. To assess the serological profiles of these two cohorts, we compared the ID_50_ values across 192 samples and measured the binding titers by semiquantitative ELISA for 199 samples, as described previously (Ng et al., 2020; O’Nions et al., 2021). There are significantly higher median immunoglobulin G (IgG) binding titers and median ID_50_ in the severe illness cohort compared with the mild illness group (Figures 3A and 3B; Figure S2), in line with previous observations (Seow et al., 2020). However, when considering how the IgG binding titer from each individual relates to the neutralization titer, it became clear that there was a discrepancy (Figures 3C and 3D). Most hospitalized individuals required a binding titer of more than 10 μg/mL to achieve strong neutralization (ID_50_ > 100). Moreover, mild infection could lead to potent neutralization (ID_50_ > 1,000) at binding titers of less than 10 μg/mL (Figure 3D), whereas this was observed for only two individuals following severe illness (Figure 3C). In fact, the amount of specific IgG present at the serum ID_50_ is significantly higher with severe illness compared with mild disease (Figure 3E).

### Effect of spike variants on mAb and serum neutralization

**Figure 4:**
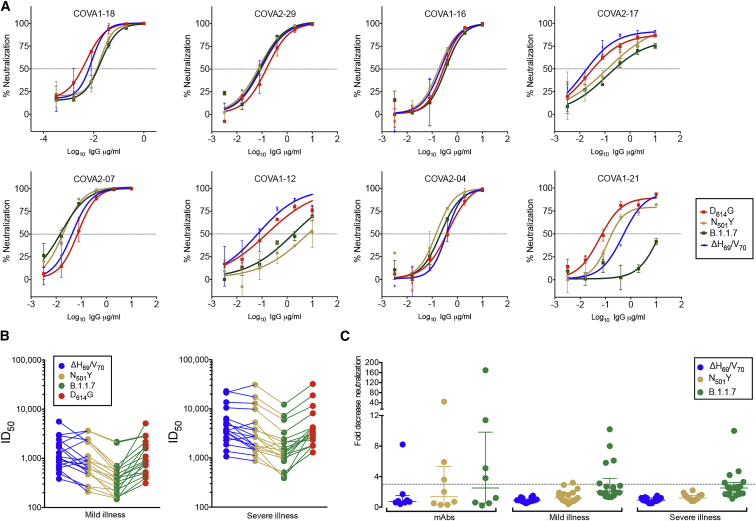
Variant B.1.1.7 SARS-CoV-2 spike effect on mAb and serum neutralization (A) The indicated mAbs were assessed by pseudotype neutralization assay. Data are representative of three independent repeats. The horizontal dotted line in each graph indicates 50% neutralization. (B) Thirty-six serum samples (mild illness, left; severe illness, right) were assessed by pseudotype neutralization assay. ID_50_ values are linked by horizontal bars for each individual sample. (C) Fold decrease in average ID_50_ values from 3 repeats for each serum sample against each mutant pseudotype versus D614G. The dotted horizontal line indicates a 3-fold drop in neutralization potency.

Investigating the ability of post-SARS-CoV-2 infection mAbs and serum to cope with mutations based on differences with SARS-CoV was a rational first approach to study escape because these mutations were likely to form viable spike proteins. However, to date, none of the mutations engineered in our study have been observed more than 20 times in global SARS-CoV-2 sequences, although other amino acid substitutions have occurred at these positions, including one change (L_452_R) that has been observed more than 1,000 times. However, additional viral variants have started to emerge on a significant scale (Li et al., 2020; Weisblum et al., 2020), such as the D_614_G mutation, observed in western Europe in February 2020 and now dominant across the globe (Korber et al., 2020). More recently, a new variant, B.1.1.7, has emerged in England and is associated with a rapid rise in case numbers (Kemp et al., 2020; Rambaut et al., 2020). B.1.1.7 encodes nine sites of change in spike relative to the original Wuhan strain. Of these, the most likely candidates to alter neutralization sensitivity are the deletion in the NTD (ΔH_69_/V_70_) and the N_501_Y substitution in the RBM (Kemp et al., 2020; Rambaut et al., 2020). Therefore, we introduced these changes into the Wuhan-strain spike in the presence of D_614_G. We found that ΔH_69_/V_70_ did not affect the neutralization potency of most of the mAbs tested, including COVA2-17 (Figure 4A), which binds the RBD and NTD (Rosa et al., 2021). The exception was the structurally unmapped COVA1-21, as reported previously (Kemp et al., 2021). Similarly, no major drop in serum neutralization was observed against ΔH_69_/V_70_ (Figure 4B). In contrast, introduction of the N_501_Y substitution dramatically lowered the neutralization potency of COVA1-12 with a fold decrease in IC_50_ of more than 40 (Figures 4A and 4C). Moreover, a 5-fold decrease in COVA2-17 potency was observed against the N_501_Y pseudotype. However, as seen for the other mutations that abrogate mAb function, the N_501_Y change had less of an effect on sera obtained after severe and mild infection (Figures 4B and 4C).

### Effect of B.1.1.7 spike on mAb and serum neutralization

Finally, a B.1.1.7 spike pseudotyping plasmid was synthesized to incorporate the mutations observed in this new variant (ΔH_69_/V_70_, ΔY_144_, N_501_Y, A_570_D, D_614_G, P_681_H, T_716_I, S_982_A, and D_1118_H). This showed that, similar to the individual N_501_Y and ΔH_69_/V_70_ mutants, B.1.1.7 can lessen the potency of three mAbs: COVA2-17, COVA1-12, and COVA1-21 (Figure 4A). These belong to distinct clusters and so do not compete for binding to the same epitope. First, COVA2-17 showed an approximate 5-fold drop in potency against the N_501_Y single mutant and the B.1.1.7 pseudotype, implying that this loss of potency is primarily N_501_Y driven. In contrast, the decrease in COVA1-12 potency noted with the single N_501_Y change was less profound against B.1.1.7. Furthermore, COVA1-21 experienced a substantial drop in potency against B.1.1.7. This mAb, which does not bind to RBD or S1 subunits, lost potency by more than 100-fold. The B.1.1.7 pseudotype was then tested against the 36 serum samples (Figures 4B and 4C). The maximum fold decrease in potency for the serum samples from mild illness was 10, but the majority of samples showed less than a 3-fold change. Similarly, the maximum decrease seen for samples from hospitalized individuals was 10-fold, but most of the samples showed a minimal change in neutralization potency. Ten samples (28%) showed a 3- to 10-fold reduction, but because they were potently neutralizing sera, the reduced ID_50_ values were still more than 1:100 with an average reduced ID_50_ of 1:523, with only two samples having an ID_50_ of less than 1:200.

## Discussion

This study demonstrates that spike mutations can diminish or abolish neutralizing activity by individual mAbs, but that serum neutralization is affected less strongly. Notably, no serum sample failed to neutralize B.1.1.7, and only one engineered mutation resulted in complete escape from neutralizing activity from just one serum sample. The spike mutants evaluated comprise seven substitutions designed to mimic possible escape changes based on homology with SARS-CoV, two observed high-frequency mutations, and the B.1.1.7 spike variant. The observation of a modest reduction in neutralization potency against B.1.1.7 by convalescent sera is consistent with concurrent reports (Collier et al., 2021; Hu et al., 2021; Shen et al., 2021). The most likely explanation for the greater effect on mAbs compared with sera is the inherent polyclonality underlying serum neutralization. This concept is supported by the observation that single spike mutations can weaken neutralization for a particular mAb but not for other mAbs in the same binding cluster. This highlights that different antibodies use distinct molecular contacts within shared epitopes so that a single mutation may not be detrimental to all antibodies in the same binding cluster. Thus, because polyclonal sera contain multiple antibodies that target the major neutralizing sites in subtly different ways, they are less sensitive to spike mutations.

The spike mutations studied here were designed to identify potential escape variants by mimicking in part the natural variation observed between SARS-CoV and SARS-CoV-2 and are focused mainly on the RBD as the major site of neutralizing antibody activity. Therefore, it was not surprising that many of the RBD-specific mAbs evaluated here lost neutralization activity against one or more of these mutations. For example, COVA2-07 and COVA2-04 lose potency against the K_417_V pseudotyped virus. COVA2-04 belongs to the VH3-53 “public” B cell receptor against SARS-CoV-2 identified from multiple human infections. Thus, COVA2-04-like antibodies are thought to be widespread among the seropositive population, but despite this, serum samples from mild infection showed very little change in neutralization potency with K_417_V pseudotyped virus. Interestingly, the strongest effect on serum samples from mild infection was mediated by the TEI_470-2_NVP substitution, which is part of what has been termed the RBD binding ridge (Greaney et al., 2021). Any mutation in this zone should be monitored closely in virus populations because of the potential for escape. Notably, the mutations that most substantially decrease serum neutralization are those that negatively affect mAb activity against the widest range of clusters (I, III, XI, IX, and VI), suggesting that mAb screening is a useful proxy for potential serum effects when a range of antibody clones is used. However, the capacity to predict the *in vivo* effect of a drop in neutralization potency requires correlation of *in vitro* serum neutralization ID_50_ values with protection, which so far has only been achieved in animal models where, encouragingly, an ID_50_ value of 1:50 was found to be protective (McMahan et al., 2021).

A caveat of the first part of this study is that only RBD substitutions were considered. Further studies to assess potential mutations before they arise should include those in the NTD, given the emerging importance of the NTD as a site for neutralizing antibodies (Andreano et al., 2020; Rosa et al., 2021). A further limitation of our original approach is that the exact mutations evaluated have not yet been found to any great degree in circulating virus populations. To understand whether the conclusions from studying the effect of the SARS-CoV-2/SARS-CoV substitutions on neutralization parallel those of real-world spike mutations, we examined the responses to the newly emerged B.1.1.7 variant (Kemp et al., 2020; Rambaut et al., 2020). The RBD mutation N_501_Y, shared between B.1.1.7, B.1.351, and P.1, did remove almost all neutralizing activity for one mAb, but, in a pattern similar to other substitutions, this did not translate into any large effect on serum potency. We did not study the changes at position 484 that have been observed in B.1.351 and P.1. Recently, pseudotyped and live B.1.351 have been shown to be resistant to neutralization by a large proportion of convalescent plasma samples (Cele et al., 2021; Wibmer et al., 2021).

Theoretically, it is likely that combinations of mutations have more potential to lead to loss of serum activity than single amino acid changes by destroying multiple parts of key epitopes. This has been observed partially in terms of the B.1.1.7 spike pseudotype analyzed here. Only one mAb was affected more dramatically by the full set of B.1.1.7 mutations compared with the ΔH_69_/V_70_ and N_501_Y individual mutations. However, serum samples with reduced neutralization were affected more strongly by B.1.1.7 (Figures 4B and 4C). Importantly, these samples were collected prior to July 2020 and therefore are highly unlikely to be derived from B.1.1.7 infection. However, all of the affected samples were still able to neutralize B.1.1.7, and the average reduced serum ID_50_ value was 1:523. This is 10 times higher than the reported serum ID_50_ correlate of protection in animal studies (McMahan et al., 2021) and suggests that these responses would likely still be effective against infection with B.1.1.7.

This study underlines the potential for escape from neutralizing antibodies because of mutations in spike and the relative resilience of serum responses compared with individual mAbs. This difference likely derives from the breadth inherent in polyclonal sera compared with the precision interaction of a given mAb. Our results suggest that the majority of vaccine responses should be effective against the B.1.1.7 variant because the sera evaluated were obtained after infection early in 2020, when the commonly circulating virus was highly similar in sequence to the vaccines now being deployed. These findings are in agreement with concurrent studies that have reported a minimal drop in neutralization potency against B.1.1.7 in vaccinee and/or convalescent sera (Muik et al., 2021; Shen et al., 2021; Wang et al., 2021b; Wu et al., 2021). Finally, because SARS-CoV-2 seropositivity will increase across the human population (because of vaccination efforts and natural infection), there may be selection for spike mutations that result in substantial antigenic drift. Recent data showing limited serum neutralization against B.1.351 (Cele et al., 2021; Wibmer et al., 2021) suggest that major antigenic drift has already occurred. Vaccine-induced responses appear to be more resilient to the mutations in B.1.351, in part because of higher initial titers (Collier et al., 2021; Wang et al., 2021a, 2021b; Wu et al., 2021). However, that this level of antigenic change has already occurred in SARS-CoV-2 suggests that, with increasing seroprevalence, additional potential neutralization escape mutations will emerge and require scrutiny. The data here suggest that evaluation of neutralizing mAbs from non-overlapping binding clusters can highlight which spike mutations will most affect serum neutralization. Our findings stress the importance of continuous monitoring of variants and *in vitro* assessment of their effect on neutralization. This is particularly relevant for use of convalescent plasma and development of therapeutic mAbs as well as vaccine development and implementation.

## Consortia

The SAFER Study Investigators are Sajida Adam, Matthew Byott, Tom Byrne, Elise Crayton, Claudia Davies, Sarah Edwards, Louise Enfield, Daniel Frampton, Kathleen Gärtner, Richard Gilson, Triantafylia Gkouleli, Nick Gotts, Andrew Hayward, Judith Heaney, Catherine F. Houlihan, Dan Lewer, Fabiana Lorencatto, Hinal Lukha, Ed Manley, Rebecca Matthews, Hazel McBain, Angela McBride, Laura E. McCoy, Carly Meyer, Susan Michie, Eleni Nastouli, Stavroula M Paraskevopoulou, Paulina Prymas, Veronica Ranieri, Emilie Sanchez, Abigail Severn, Maryam Shahmanesh, Robert Shortman, Moira J. Spyer, Andrea Stoltenberg, Nina Vora, Naomi Walker, Bethany Williams, Jared Wilson-Aggarwal, and Leigh Wood.

## STAR★Methods

### Key resources table

**Table:** 

REAGENT or RESOURCE	SOURCE	IDENTIFIER
**Antibodies**		

Goat anti-human F(ab)’2	Stratech	Cat# 109-006-006; RRID: AB_2337553
Alkaline phosphatase-conjugated goat anti-human IgG	Stratech	Cat#109-055-098; RRID: AB_2337608

**Bacterial and virus strains**		

XL1-Blue Supercompetent Cell	Agilent	Cat# 210518

**Biological Samples**		

Mild illness serum samples	UCLH SAFER study; Houlihan et al., 2020	NHS Health Research Authority reference no. 20/SC/0147
Severe illness patient serum samples	Tissue Access for Patient Benefit (TAPb), The Royal Free Hospital	Reference no. NC2020.24; NRES EC no. 16/WA/0289

**Chemicals, peptides, and recombinant proteins**		

SARS-CoV-2 spike S1 protein	Peter Cherepanov Laboratory; Ng et al., 2020	N/A

**Critical commercial assays**		

Bright-Glo Luciferase kit	Promega	Cat# E2650
QuickChange Lightening Site-Directed Mutagenesis kit	Agilent	Cat# 210518

**Experimental models: cell lines**		

Human: HEK293T/17 cells	American Type Culture Collection	ATCC CRL-11268
Human: HeLa-ACE-2 cells	James Voss Laboratory, The Scripps Research Institute; Rogers et al., 2020	N/A

**Oligonucleotides**		

5′-agcaatttcagagtgcagcctaccgGg GAcatcgtgagattccctaatatcacc-3′	ES_324-6_GD Forward	1107
5′-tacagcgtgctgtacaatagcg ccTTcttcagcaccttcaaatgttatggt-3′	S_373_F Forward	1109
5′-accttcaaatgttatggtgtttcgGcaa caaagctgaatgacctgtgcttc-3′	P_384_A Forward	1111
5′-cagatcgcgccagggcagaccgg cGTgatcgccgactacaattacaagctg-3′	K_417_V Forward	1112
5′-tggaactctaacaatctagattcgaCa TCtACaggcaattacaattacctgtacaga-3′	KVG_444-6_TST Forward	1114
5′-aaagttggaggcaattacaattacA Agtacagactgttcagaaagagcaat-3′	L_452_K Forward	1115
5′-ggcaattacaattacctgtacagaTA CCtcagaaagagcaatctgaagcctttc-3′	LF_455-6_YL Forward	1116
5′-aagcctttcgagagagacatcagcaAcgTg CCctaccaggccggcagcacaccgtgt-3′	TEI_470-472_NVP Forward	1118
5′-ttcaattgctacttccctctgcag GActacggcttccagcctaccaatggc-3′	S_494_D Forward	1122
5′-ggtgatattagggaatctcacgatg TCcCcggtaggctgcactctgaaattgct-3′	ES_324-6_GD Reverse	1126
5′-accataacatttgaaggtgctgaagAAg gcgctattgtacagcacgctgta-3′	S_373_F Reverse	1128
5′-gaagcacaggtcattcagctttg ttgCcgaaacaccataacatttgaaggt-3′	P_384_A Reverse	1130
5′-cagcttgtaattgtagtcggcga tcACgccggtctgccctggcgcgatctg-3′	K_417_V Reverse	1131
5′-tctgtacaggtaattgtaattgcctGTa GAtGtcgaatctagattgttagagttcca-3′	KVG_444-6_TST Reverse	1133
5′-attgctctttctgaacagtctgtacTTgta attgtaattgcctccaacttt-3′	L_452_K Reverse	1134
5′-gaaaggcttcagattgctctttctgaGGTA tctgtacaggtaattgtaattgcc-3′	LF_455-6_YL Reverse	1135
5′-acacggtgtgctgccggcctggta gGGcAcgTtgctgatgtctctctcgaaaggctt-3′	TEI_470-472_NVP Reverse	1137
5′-gccattggtaggctggaagccgtag TCctgcagagggaagtagcaattgaa-3′	S_494_D Reverse	1141

**Recombinant DNA**		

pCDNA3.1+ D614G spike expression vector	Kemp et al., 2021	pCDNA_Spike D_614_G
pCDNA3.1+ D614G_ΔH_69_/V_70_ Spike expression vector	Kemp et al., 2021	pCDNA_Spike D_614_G_ΔH_69_/V_70_
pCDNA3.1+ D614G_N501Y spike expression vector	Generated in this study	pCDNA_Spike D_614_G_N_501_Y
SARS-CoV-2 Wuhan spike pCDNA3.1+ expression vector	Seow et al., 2020	pCDNA_Spike
pCDNA3.1+ B.1.1.7 spike (ΔH69/V70, ΔY144, N501Y, A570D, D614G, P681H, T716I, S982A, D1118H) expression vector	Synthesized by Genewiz Inc. and subcloned into pcDNA3.1+	pCDNA_B.1.1.7
SARS-CoV-2 Wuhan spike pCDNA3.1+ expression vector P384A mutation	Generated in this study	P_384_A
SARS-CoV-2 Wuhan spike pCDNA3.1+ expression vector K417V mutation	Generated in this study	K_417_V
SARS-CoV-2 Wuhan spike pCDNA3.1+ expression vector KVG446-TST mutation	Generated in this study	KVG_444-6_TST
SARS-CoV-2 Wuhan spike pCDNA3.1+ expression vector L452K mutation	Generated in this study	L_452_K
SARS-CoV-2 Wuhan spike pCDNA3.1+ expression vector LF455-6YL mutation	Generated in this study	LF_455-6_YL
SARS-CoV-2 Wuhan spike pCDNA3.1+ expression vector TEI470-2NVP mutation	Generated in this study	TEI_470-472_NVP
SARS-CoV-2 Wuhan spike pCDNA3.1+ expression vector S494D mutation	Generated in this study	S_494_D
SARS-CoV-2 Wuhan spike pCDNA3.1+ expression vector ES324-6GD mutation	Generated in this study	ES_324-6_GD
SARS-CoV-2 Wuhan spike pCDNA3.1+ expression vector S373F mutation	Generated in this study	S_373_F
HIV-1 luciferase reporter vector	Seow et al., 2020	CSWL HIV-1 luciferase reporter
HIV p8.91 packaging construct	Zufferey et al., 1997	p8.91

**Software and Algorithms**		

UCSF Chimera	Pettersen et al., 2004	https://www.cgl.ucsf.edu/chimera/
Prism 8	GraphPad prism	https://www.graphpad.com/scientific-software/prism/

### Resource availability

#### Lead contact

Further information and requests for resources and reagents should be directed to and will be fulfilled by the Lead Contact, Laura McCoy (l.mccoy@ucl.ac.uk).

#### Materials availability

Reagents generated in this study are available from the Lead Contact with a completed Materials Transfer Agreement.

#### Data and code availability

This study did not generate datasets/code. Original source data for SARS-CoV-2 spike structure used in Figure 2 is available at https://doi.org/10.2210/pdb7DF4/pdb, and in Figure S1 at https://doi.org/10.2210/pdb6VXX/pdb.

### Experimental model and subject details

#### Mild illness serum samples

These samples are part of the UCLH SAFER study and were collected as previously described (Houlihan et al., 2020). Briefly, samples are from 81 seropositive individuals previously identified (Houlihan et al., 2020) who donated blood at monthly intervals from March to July 2020 as well as undergoing regular PCR testing. Informed consent was obtained from all participants. The median age of participants was 81 (interquartile range 70-87), 43% were female and 57% male. The study protocol was approved by the NHS Health Research Authority (ref 20/SC/0147) on 26 March 2020. Ethical oversight was provided by the South- Central Berkshire Research Ethics Committee.

#### Severe illness serum samples

These samples are from patients hospitalized for COVID-19 between March and July 2020 and were obtained during their hospital stay through the Tissue Access for Patient Benefit (TAPb) scheme at The Royal Free Hospital (approved by UCL–Royal Free Hospital BioBank Ethical Review Committee Reference number: NC2020.24 NRES EC number: 16/WA/0289). Informed consent was obtained from all participants and a single blood sample was taken without interfering with normal clinical care. The median age of participants was 34 years (interquartile range 29–44), 62% were female and 38% male.

#### Bacterial Strains and Cell Culture

Bacterial transformations were performed with XL1-Blue Supercompetent Cells (Agilent). SARS-CoV-2 pseudotypes were produced by transfection of HEK293T/17 cells and neutralization activity assayed using HeLa cells stably expressing ACE2 (Kind gift James E Voss).

### Method details

#### Spike mutant generation

QuikChange Lightening Site-Directed Mutagenesis kit was used to generate amino acid substitutions in the SARS-CoV-2 Wuhan spike expression vector (Seow et al., 2020) or the D614G pCDNA spike plasmid (Kemp et al., 2021) following the manufacturer’s instructions (Agilent Technologies, Inc., Santa Clara, CA). Spike B.1.1.7 (ΔH_69_/V_70_, ΔY_144_, N_501_Y, A_570_D, D_614_G, P_681_H, T_716_I, S_982_A, D_1118_H) was synthesized by Genewiz, Inc. and cloned into the pCDNA3.1+ expression vector using BamHI and EcoRI restriction sites.

#### Neutralization assay

HIV-1 particles pseudotyped with SARS-CoV-2 spike were produced in a T75 flask seeded the day before with 3 million HEK293T/17 cells in 10 ml complete DMEM, supplemented with 10% FBS, 100 IU/ml penicillin and 100 μg/ml streptomycin. Cells were transfected using 60 μg of PEI-Max (Polysciences) with a mix of three plasmids: 9.1  μg HIV-1 luciferase reporter vector (Seow et al., 2020), 9.1  μg HIV p8.91 packaging construct and 1.4  μg WT SARS-CoV-2 spike expression vector (Seow et al., 2020). Supernatants containing pseudotyped virions were harvested 48 h post-transfection, filtered through a 0.45-μm filter and stored at −80°C. Neutralization assays were conducted by serial dilution of monoclonal IgG at the indicated concentrations in DMEM (10% FBS and 1% penicillin–streptomycin) and incubated with pseudotyped virus for 1 h at 37°C in 96-well plates. HeLa cells stably expressing ACE-2 (provided by J.E. Voss, Scripps Institute) were then added to the assay (10,000 cells per 100 μl per well). After 48-72 h luminescence was assessed as a proxy of infection by lysing cells with the Bright-Glo luciferase kit (Promega), using a Glomax plate reader (Promega). Measurements were performed in duplicate and used to calculate 50% inhibitory dilutions/concentration (ID/C_50_) values in GraphPad Prism software.

#### Semiquantitative ELISA

As described previously (O’Nions et al., 2021) nine columns of a half-well 96-well MaxiSorp plate were coated with purified SARS-CoV-2 spike S1 protein in PBS (3 μg/ml per well in 25 μL) and the remaining three columns were coated with 25 μL goat anti-human F(ab)’2 diluted 1:1000 in PBS to generate the internal standard curve. After incubation at 4°C overnight, the ELISA plate was blocked for 1 h in assay buffer (PBS, 5% milk, 0.05% Tween 20). Sera was diluted in assay buffer at dilutions from 1:50 to 1:5000 and 25 μL added to the ELISA plate. Serial dilutions of known concentrations of IgG standards were applied to the three standard curve columns in place of sera. The ELISA plate was then incubated for 2 h at room temperature and then washed 4 times with PBS-T (PBS, 0.05% Tween 20). Alkaline phosphatase-conjugated goat anti-human IgG at a 1:1000 dilution was then added to each well and incubated for 1 h. Following this, plates were washed 6 times with PBS-T and 25 μL of colorimetric alkaline phosphatase substrate added. Absorbance was measured at 405 nm. Antigen-specific IgG concentrations in serum were then calculated based on interpolation from the IgG standard results using a four-parameter logistic (4PL) regression curve fitting model.

### Quantification and statistical analysis

All neutralization measurements were performed in duplicate and 50% inhibitory concentrations/dilutions (IC/ID_50_) were calculated using GraphPad Prism software. ID_50_ values calculated as indicated in the relevant Figure legends. Statistical analysis in Figure 3 (non-parametric Mann-Whitney U test) was performed using GraphPad Prism software, significance defined as ∗∗∗∗p < 0.05. Fold decrease in serum ID_50_ was calculated by dividing the average ID_50_ value for a given sample against SARS-CoV-2 or SARS-CoV-2 D_614_G (as indicated) by the average ID_50_ value for that sample against the indicated mutant or variant pseudotype. Fold decrease in mAb IC_50_ was calculated by dividing the average IC_50_ value for a given mAb against the indicated mutant or variant pseudotype by the average IC_50_ value for that mAb against the SARS-CoV-2 or SARS-CoV-2 D_614_G (as indicated).

## Supplemental information

Document S1. Figures S1 and S2

Document S2. Article plus supplemental information
