# Supplementary material for: The effect of spike mutations on SARS-CoV-2 neutralization
Source: Cell Rep. 2021 Mar 6;34(12):108890. doi: 10.1016/j.celrep.2021.108890 (PMC7936541; doi:10.1016/j.celrep.2021.108890)
Supplement: Document S1. Figures S1 and S2 [file mmc1.pdf]

Cell Reports, Volume *34*

Supplemental information

The effect of spike mutations on SARS-CoV-2 neutralization

Chloe Rees-Spear, Luke Muir, Sarah A. Griffith, Judith Heaney, Yoann Aldon, Jonne L. Snitselaar, Peter Thomas, Carl Graham, Jeffrey Seow, Nayung Lee, Annachiara Rosa, Chloe Roustan, Catherine F. Houlihan, Rogier W. Sanders, Ravindra K. Gupta, Peter Cherepanov, Hans J. Stauss, Eleni Nastouli, on behalf of the SAFER Investigators, Katie J. Doores, Marit J. van Gils, and Laura E. McCoy

Supplemental Information

Figure S1. Substitution in SARS-CoV-2 based on SARS-CoV sequence, related to Figure 1.
Figure S2. Binding and neutralization titers across two infection cohorts, related to Figure 3.

Figure S1. Substitution in SARS-CoV-2 based on SARS-CoV sequence, related to Figure 1.

(A) Alignment of RBD from pseudotype SARS-CoV-2 Spike plasmid with SARS-CoV consensus amino acids. Sequences were aligned using the Clustal Omega (1.2.4) tool. The amino acid positions removed in the Spike plasmid are highlighted in grey and the residues of SARS-CoV that replaced them are indicated in red text. Amino acid positions for the first residue changed in each individual mutant is positioned above the first mutated residue in any given mutant. A ¶ by the residue number indicates the pseudotyped virus carrying this substitution did not yield sufficiently infectious titre for analysis of antibody sensitivity. The RBM region in both sequences is underlined. Conservation symbols are listed below the aligned residues and are as follows: an * (asterisk) indicates positions that have a single, fully conserved residue; a : (colon) indicates conservation between groups of strongly similar properties; a . (period) indicates conservation between groups of weakly similar properties; a (gap) indicates no conservation.
(B) SARS CoV-2 RBD (PDB code 6VXX) showing WT (top) and mutated (bottom) amino acid side chains at residues of interest. Using UCSF Chimera, amino acid side chains were altered at select positions using Dunbrack 2010 backbone-dependent rotamer library (Shapovalov & Dunbrack, 2011). Note that the image does not show structure alterations that may occur as a result of mutated residues.
(C) Each SARS-CoV-2 pseudotype mutant is listed on the left and the mAb clusters are shown on the right. Mutations that result in a complete loss of function are indicated by a line joining the mutant to the mAb. Mutations that result in a partial loss of function are indicated by a dashed line joining the mutant to the mAb. Two mutations that had minimal/no effect on function are not linked to any mAb.

Shapovalov, M. V., & Dunbrack, R. L., Jr. (2011). A smoothed backbone-dependent rotamer library for proteins derived from adaptive kernel density estimates and regressions. *Structure, 19*(6), 844-858. doi:10.1016/j.str.2011.03.019

A

```
                                                 324                                 346
pcDNA-SARS-CoV2 Spike   SETKCTLKSFTVEKGIYQTSNFRVQPTESIVRFPNITNLCPFGEVFNATRFASVYAWNRK
SARS-CoV-consensus      AELKCSVKSFEIDKGIYQTSNFRVVPSGDVVRFPNITNLCPFGEVFNATKFPSVYAWERK
                        :* **::*** ::*********** *: .:*******************:* *****:**
                                          373        384
pcDNA-SARS-CoV2 Spike   RISNCVADYSVLYNSASFSTFKCYGVSPTKLNDLCFTNVYADSFVIRGDEVRQIAPGQTG
SARS-CoV-consensus      KISNCVADYSVLYNSTFFSTFKCYGVSATKLNDLCFSNVYADSFVVKGDDVRQIAPGQTG
                        :**************: ********** ********:********::**:**********
                       417                    439¶ 444      452 455 459       470 474¶
pcDNA-SARS-CoV2 Spike   KIADYNYKLPDDFTGCVIAWNSNNLDSKVGGNYNYLYRLFRKSNLKPFERDISTEIYQAG
SARS-CoV-consensus      VIADYNYKLPDDFMGCVLAWNTRNIDATSTGNYNYKYRYLRHGKLRPFERDISNVPFSPD
                         ************ ***:***:.*:*:.  ***** ** :*:.:*:*******.  :. .
                       477  481¶      494
pcDNA-SARS-CoV2 Spike   STPCNGVEGFNCYFPLQSYGFQPTNGVGYQPYRVVVLSFELLHAPATVCGPKKSTNLVKN
SARS-CoV-consensus      GKPCTP-PALNCYWPLNDYGFYTTTGIGYQPYRVVVLSFELLNAPATVCGPKLSTDLIKN
                        ..**.   .:***:**:.***  *.*:****************:*********  **:*:**
```

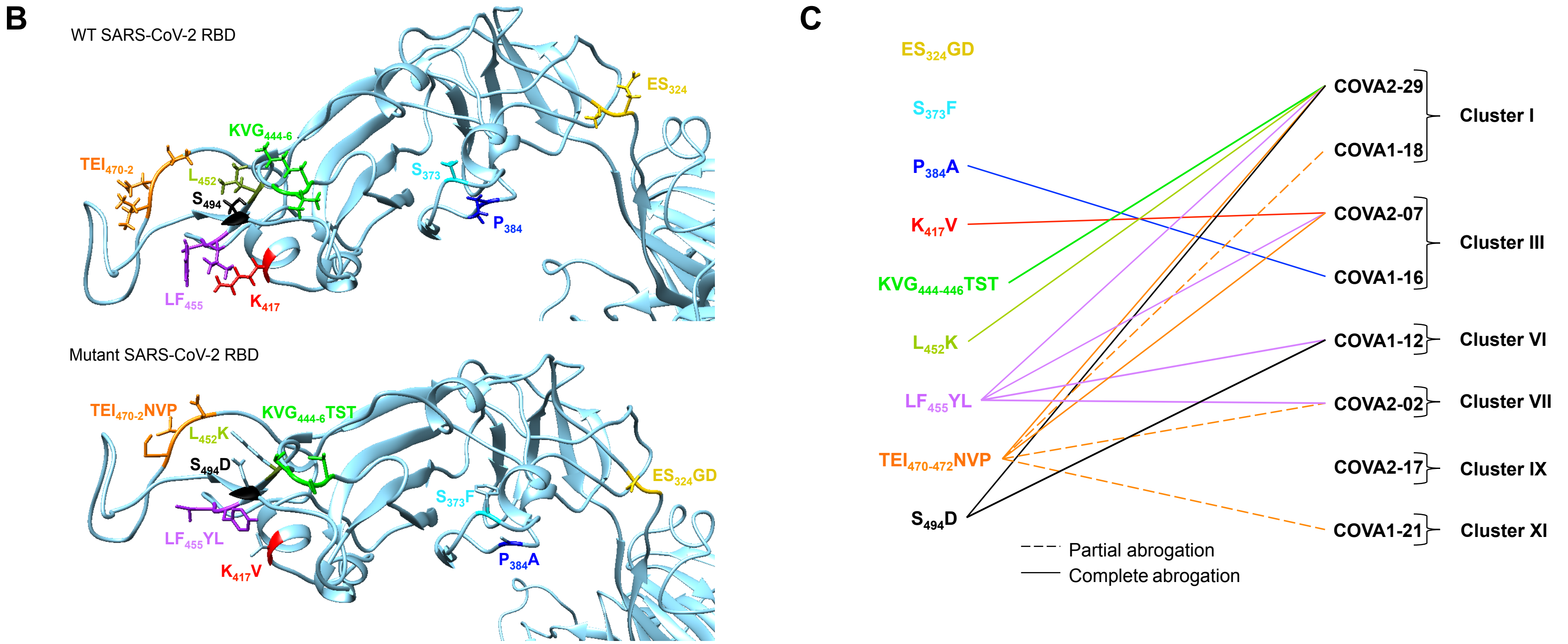

Figure S2. Binding and neutralization titers across two infection cohorts, related to Figure 3.

Binding and neutralization titers were generated as described in the Methods. Concentrations of S1-specific serum IgG (pg) at ID_{50} dilutions were calculated using the IgG titers quantified via the semi-quantitative ELISA and the known ID_{50} value. Only sera that gave a measurable titer in both semi-quantitative ELISA and pseudotype neutralization assay were included. Column statistics below were generated in Prism Graphpad and the data were analyzed by a non-parametric Mann-Whitney U test.

	Binding titer (S1 IgG µg/ml)		Neutralization titer (ID_{50})		Specific IgG at ID_{50}	
	Mild	Severe	Mild	Severe	Mild	Severe
**n =**	105	94	99	93	92	91
**Minimum**	0.3	2	50	50	12.86	68.06
**25% Percentile**	1.6	17	201.3	513.8	253.9	971.3
**Median**	3.9	46.5	342.3	1466	558.6	1757
**75% Percentile**	7.2	122.8	666.4	2556	1155	3073
**Maximum**	68	529	4152	20601	2907	45053
**Mean**	5.562	89.56	603.8	2347	736.5	3181
**Std. Deviation**	7.429	108.2	718.5	3008	604.7	5540
**Std. Error of Mean**	0.725	11.16	72.21	311.9	63.04	580.8
**Lower 95% CI of mean**	4.124	67.4	460.5	1727	611.3	2027
**Upper 95% CI of mean**	6.999	111.7	747.1	2966	861.7	4335

	Mann Whitney test	Mann Whitney test	Mann Whitney test
**P value**	< 0.0001	< 0.0001	< 0.0001
**Exact or approximate P value?**	Exact	Exact	Exact
**P value summary**	****	****	****
**Significant (P < 0.05)**	Yes	Yes	Yes
**One- or two-tailed P value?**	Two-tailed	Two-tailed	Two-tailed
**Sum of ranks in column A,B**	6217, 13684	6862, 11667	5777, 11059
**Mann-Whitney U**	651.5	1912	1499
